# Growth of Graphene/h-BN Heterostructures on Recyclable Pt Foils by One-Batch Chemical Vapor Deposition

**DOI:** 10.1038/s41598-017-17432-9

**Published:** 2017-12-06

**Authors:** Yongteng Qian, Huynh Van Ngoc, Dae Joon Kang

**Affiliations:** Department of Physics and Institute of Basic Sciences, Sungkyunkwan University, 2066, Seobu-ro, Jangan-gu, Suwon, 16419 Gyeonggi-do Republic of Korea

## Abstract

High-quality large-area graphene/h-BN vertical heterostructures are promising building blocks for many viable applications such as energy harvesting/conversion, electronics and optoelectronics. Here, we successfully grew high-quality large-area graphene/h-BN vertical heterostructures on Pt foils by one-batch low-pressure chemical vapor deposition (LPCVD). We obtained the high quality of about 200-µm-wide graphene/h-BN film having uniform layer thickness. Moreover, the obtained graphene/h-BN heterostructures exhibited field effect mobility of up to 7,200 cm^2^V^−1^s^−1^ at room temperature. These results suggest that such graphene/h-BN heterostructures on recyclable Pt foils grown by LPCVD are promising for high-performance graphene-based electronics.

## Introduction

Vertical heterostructures based on 2 dimensional (2D) layered materials such as graphene and h-BN have emerged as a new paradigm of functional materials^1,2^. Representing the thinnest and the most common 2D heterostructures, graphene/h-BN, have recently attracted great attentions due to their remarkable morphological, electrical, and thermal properties^3–5^. Such heterostructures have stimulated extensive interest for exploring some novel physics issues, such as fractional quantum Hall effect^6^, commensurate-incommensurate transitions^7^, Hofstadter’s butterfly behaviors^8^, and ballistic transport^9^. Thus, securing reliable methods for producing large-area, high-quality graphene/h-BN heterostructures becomes the most significant step for related fields. One plausible strategy for producing graphene/h-BN vertical heterostructures is based on layer-by-layer transfer of separately exfoliated 2D layers via a two-step process^10,11^. However, such a two-step process has some drawbacks during the transfer process, such as unwanted charge trapping that originates from any transfer-induced contaminants and defects, and poor surface flatness during the transfer; all of these are undesirable for obtaining high quality graphene/h-BN vertical heterostructures^12,13^. These drawbacks negatively affect the electrical and optical characteristics of graphene. Up to now, there have been some attempts for a direct CVD-based growth method of graphene on h-BN films to overcome these outstanding issues. Direct CVD-based growth of graphene on h-BN films may enable not only to obtain large-area uniform graphene films but also to reliably form versatile graphene/h-BN vertical heterostructures^14–16^. However, this approach still suffers from many technical issues such as poor quality of h-BN films, small size of graphene grains on h-BN, and h-BN film degradation that is caused by etching by H- and/or O-containing gaseous species during the growth of graphene^2,17^.

In this work, we successfully grew continuous, large-area graphene/h-BN vertical heterostructures on recyclable Pt foils of 6 mm × 25 mm using one-batch LPCVD method. The growth process was implemented by employing a recyclable Pt foil and CH_4_ gas as the substrate and the carbon precursor carrier, respectively. We found that using a single layer h-BN film as the substrate allows us to obtain graphene/h-BN heterostructures, because direct growth of graphene on h-BN can retain the pristine properties of graphene. Furthermore, facile transfer of graphene/h-BN vertical heterostructures (directly grown using the LPCVD method) onto arbitrary substrates via a straightforward electrochemical bubbling-based release method presents a unique recipe for fabricating many viable devices^18^. Our transfer method provides an additional advantage, *i.e*., it is characterized by a rapid process and extended use of recyclable Pt foils. The field effect mobility of the fabricated graphene/h-BN heterostructures based field effect transistors is as high as 7,200 cm^2^V^−1^s^−1^ at room temperature, suggesting that our approach of directly growing graphene/h-BN heterostructures on Pt foils using LPCVD can be quite promising for high-performance graphene-based electronics.

## Results and Discussion

### LPCVD growth of graphene on h-BN/Pt foil and electrochemical bubbling method transfer of graphene/h-BN film from Pt foil

**Figure 1:**
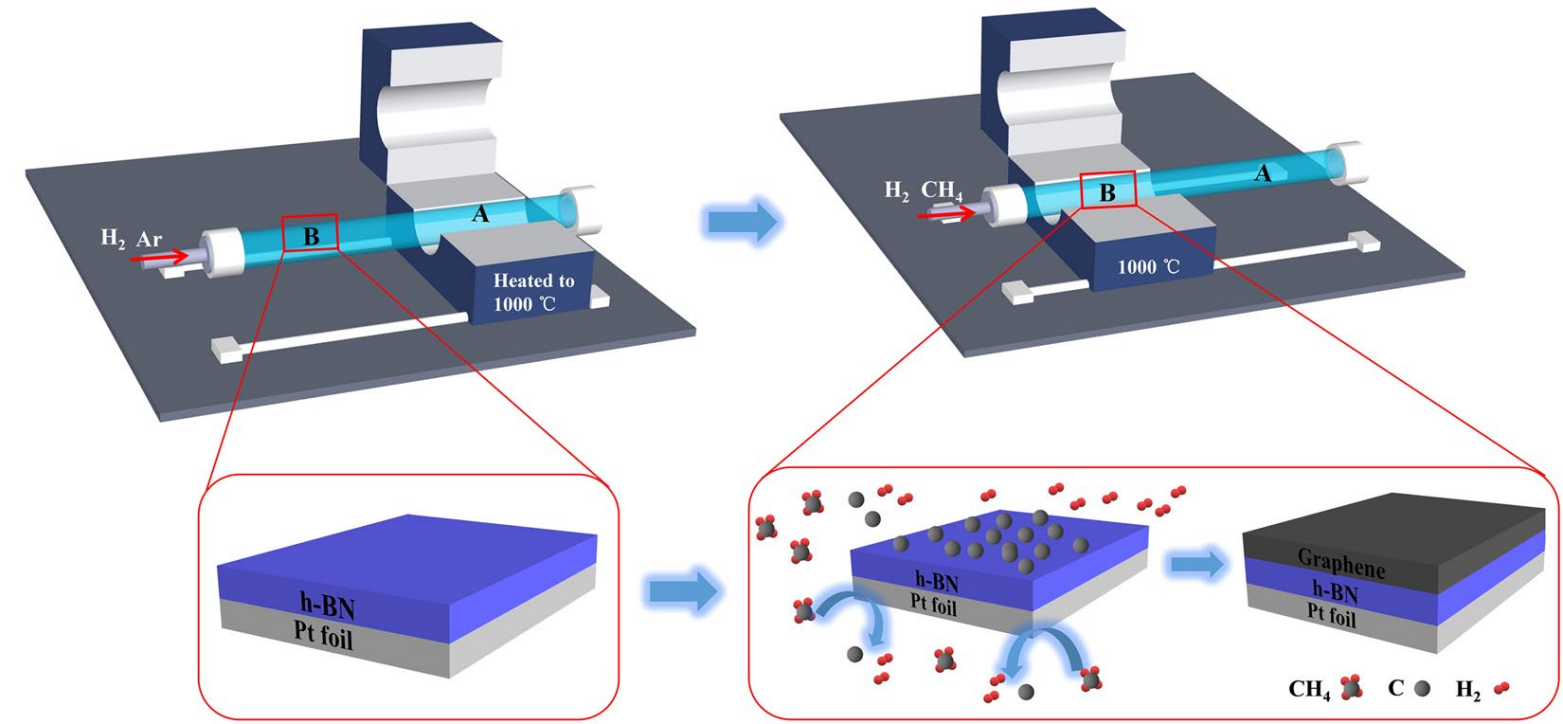
Schematic of the thermal CVD system for growing single-layer graphene on a h-BN/Pt foil.

**Figure 2:**
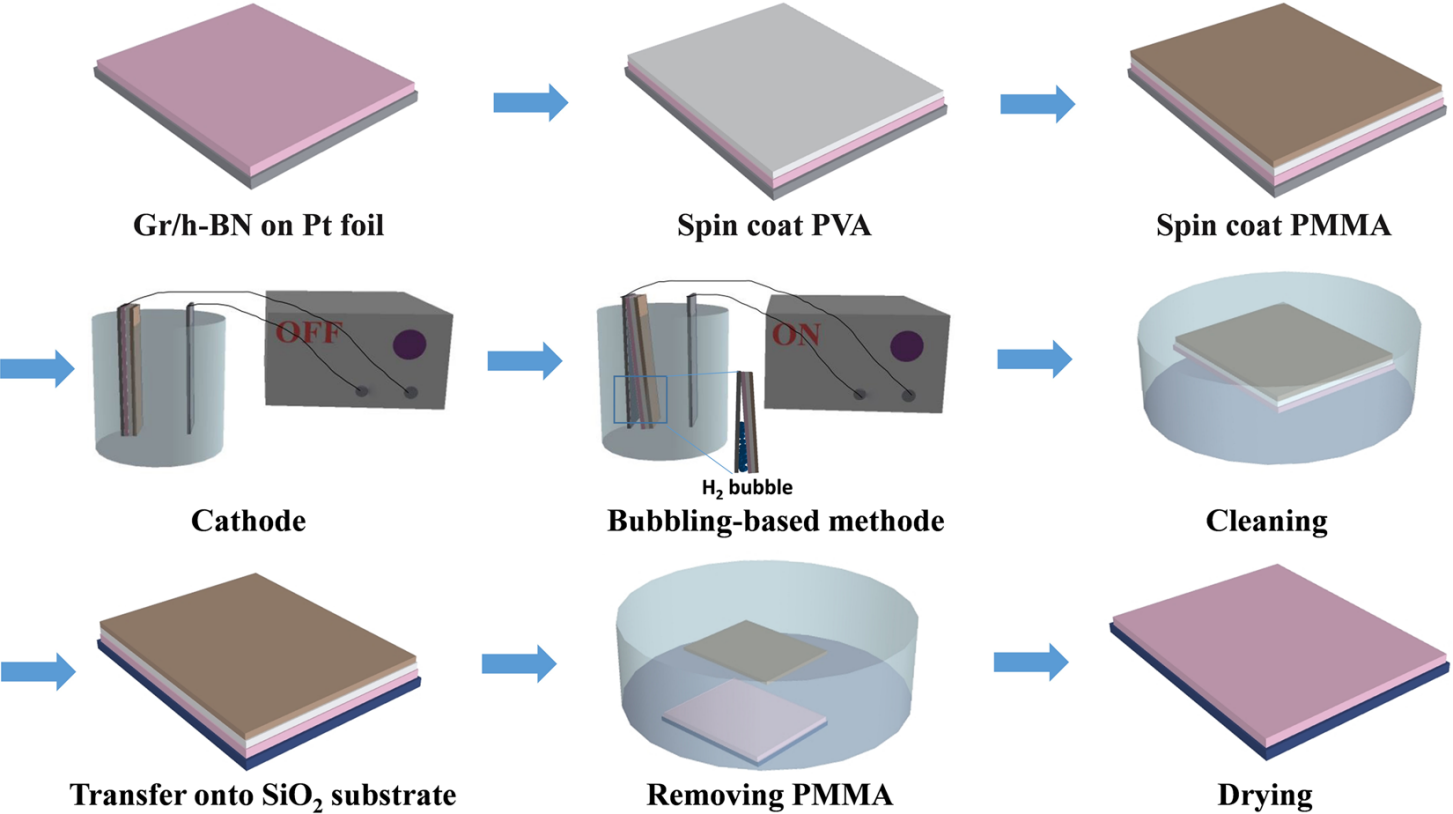
Schematic of the method for transferring graphene/h-BN onto a 300-nm-thick SiO_2_/Si substrate using the electrochemical bubbling transfer method.

Figures 1 and 2 show the schematic of the LPCVD system for growing single-layer graphene on h-BN/Pt foils and electrochemical bubbling method-based transfer of a graphene/h-BN film onto a 300-nm-thick SiO_2_/Si substrate, respectively (more details are provided in the Methods section).

### Optical micrographs and Raman spectra

**Figure 3:**
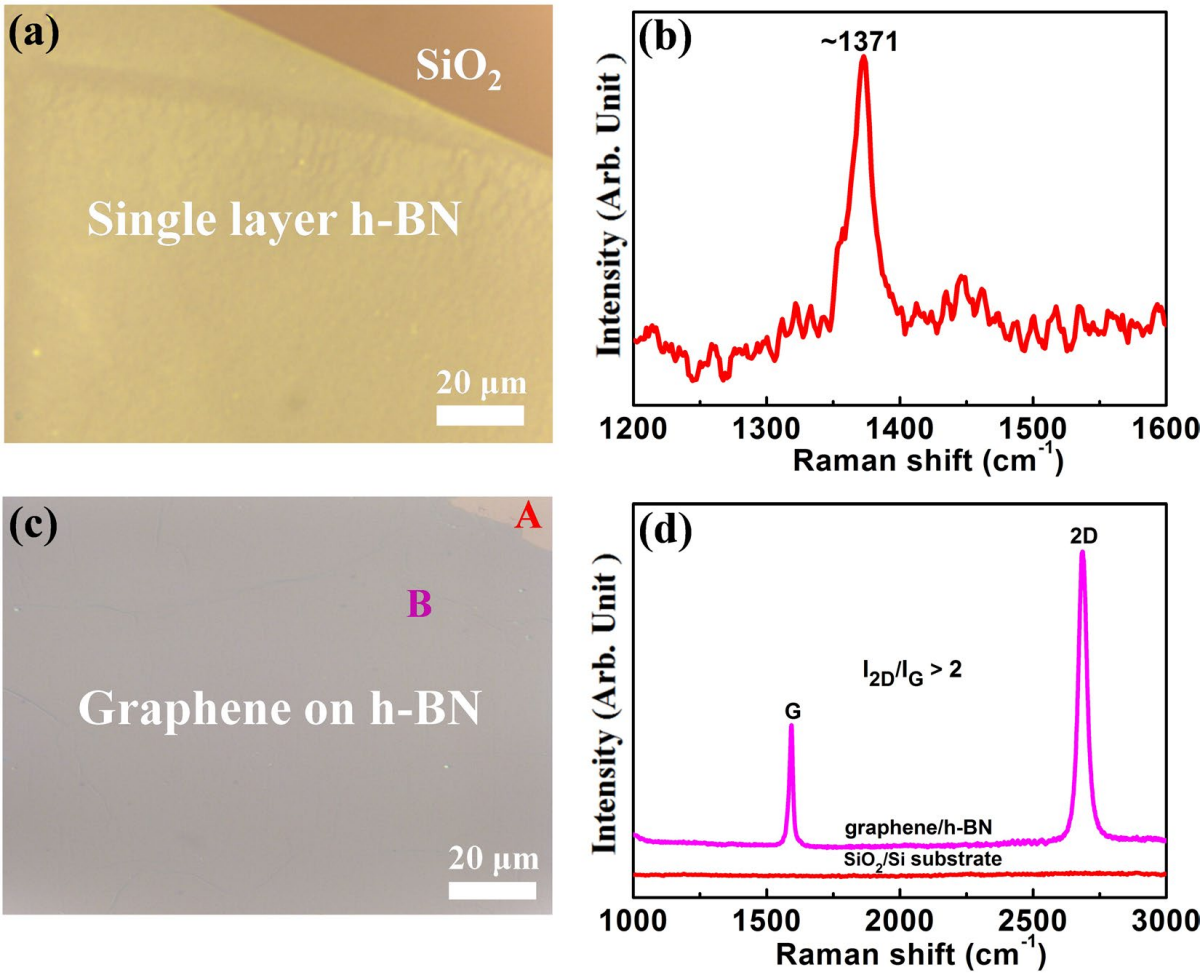
Optical images and Raman spectra of h-BN and graphene/h-BN transferred onto a 300-nm-thick SiO_2_/Si substrate. 3(**b**) Raman spectrum of single-layer h-BN in (**a**). (**d**) Raman spectra of graphene/h-BN and SiO_2_/Si substrate in (**c**), where A represents the region of the SiO_2_/Si substrate and B is the region of graphene/h-BN.

**Figure 4:**
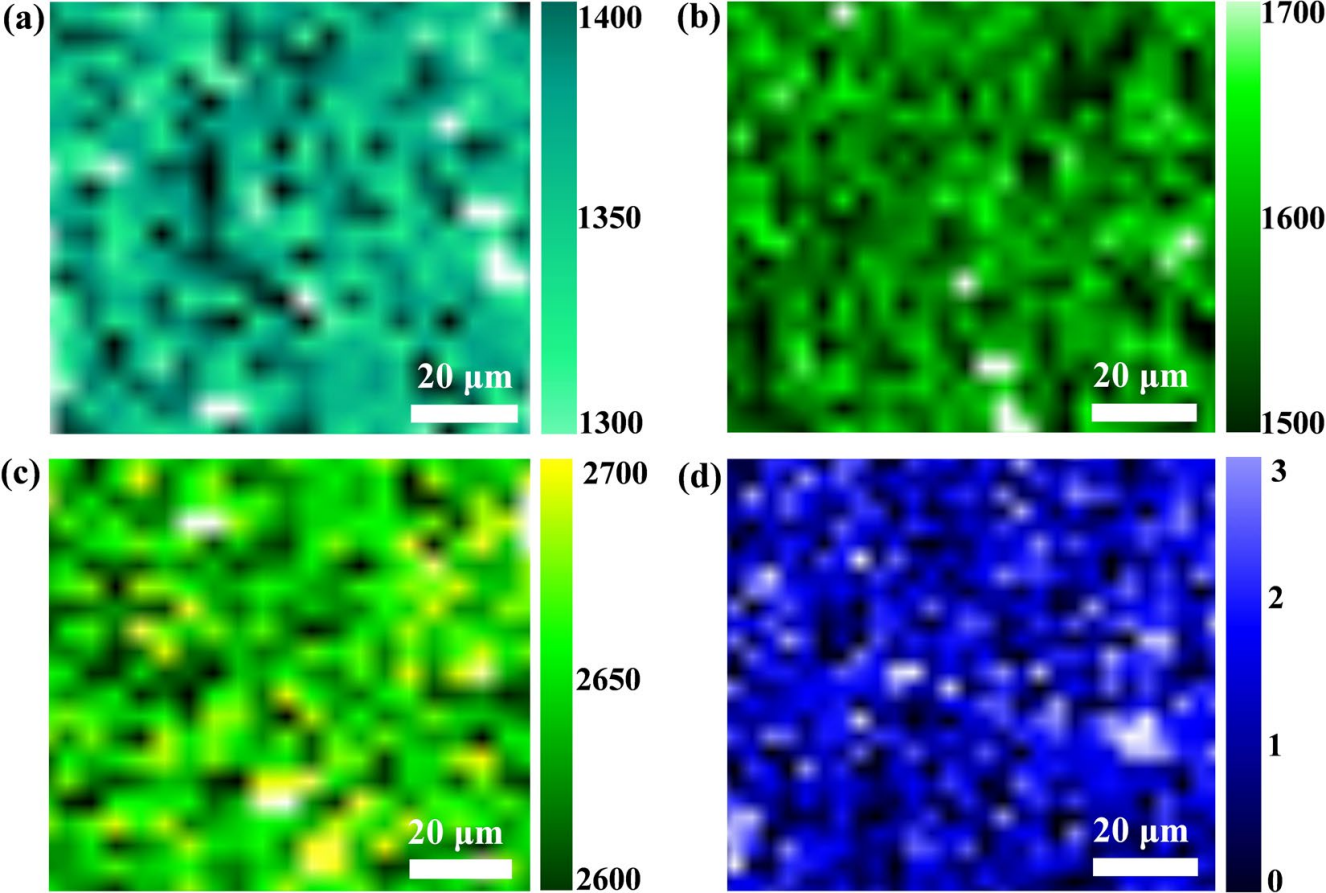
Raman mappings of graphene/h-BN heterostructures: Raman mappings of (**a**) h-BN peak; (**b**) G peak; (**c**) 2D peak; (**d**) I_2D_/I_G_.

Optical microscopy and Raman spectroscopy were used for determining the surface features and the structural quality of h-BN and graphene/h-BN films. Figure 3(a),(b) show the optical image and the Raman spectrum of the h-BN film. Figure 3(a) reveals that the surface of the h-BN film is continuous without impurities and wrinkles, suggesting a high-quality h-BN film. Figure 3(b) reveals that the Raman spectrum of the h-BN film exhibits a peak at ~1,371 cm^−1^, demonstrating that the h-BN film is a single layer^19,20^. Figure 3(c) suggests that, in terms of optical inspection, the graphene/h-BN film on the SiO_2_/Si substrate is continuous and large area. Figure 3(d) shows the Raman spectrum of the graphene/h-BN film transferred onto the SiO_2_/Si substrate along with the Raman spectrum of a bare SiO_2_/Si substrate. The two locations A and B, representing the regions of the bare SiO_2_/Si substrate and the graphene/h-BN film respectively as in Fig. 3(c), were chosen for comparison, and the corresponding Raman spectra are shown in Fig. 3(d) in red and pink respectively. While the red spectrum reveals no peaks, the pink spectrum features clear G and 2D peaks of graphene at 1,600 cm^−1^ and 2,690 cm^−1^, respectively. The I_2D_/I_G_ peak intensity ratio was well over 2, and the full width at half maximum (FWHM) of the 2D band was estimated to be 37 cm^−1^. This observation clearly indicates that the graphene film is a single-layer^21^. The scanning Raman mappings of the h-BN peak, G peak, 2D peak of the graphene and I_2D_/I_G_ intensity ratio over a 75 μm × 75 μm area with a spot size of 2 μm and a step size of 2 μm were obtained, as shown in Fig. 4. Figure 4 (a) shows that the Raman mapping of h-BN peak regions indicated the uniform of h-BN. Moreover, the Raman mapping of the G peak (the range from 1580 to 1600 cm^−1^), 2D peak (the range from 2670–2690 cm^−1^), as well as I_2D_/I_G_ intensity ratio (was close to 2), also revealed that the graphene film was grown on h-BN film (Fig. 4(b–d)).

**Figure 5:**
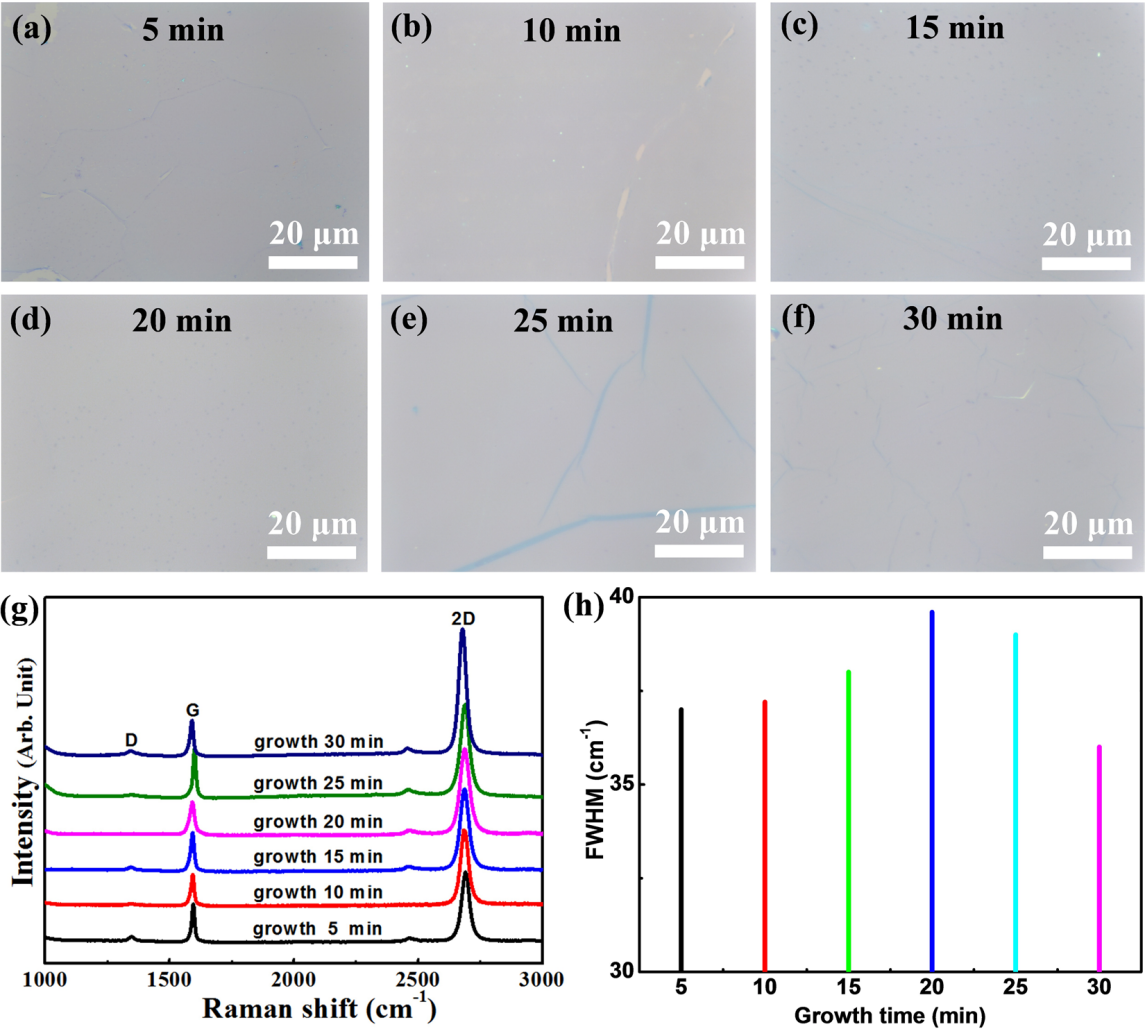
(**a**–**f**) Optical images of graphene on h-BN, for growth times of 5, 10, 15, 20, 25, and 30 min, respectively. (**g**) Raman spectra and (**h**) FWHM of the 2D band of graphene on h-BN, for growth times of 5, 10, 15, 20, 25, and 30 min.

Figure 5(a–f) show the optical images of the graphene/h-BN film grown on the Pt foil, for different growth times. When the growth time was under 10 min, poor-quality graphene on the h-BN was obtained, and on visual inspection, the surface of graphene was found to be discontinuous (Fig. 5(a),(b)). For the growth time of 15 min, the surface of the graphene/h-BN film was continuous, but some particulate defects were observed (Fig. 5(c)). For the growth time of 20 min, the surface of the graphene/h-BN film was clearly continuous over a large area without any appreciable defects (Fig. 5(d)). For longer growth times (25 min and 30 min), the surface of the resulting graphene/h-BN film featured some wrinkles (Fig. 5(e),(f)). Based on these observations, we concluded that the optimal growth time is 20 min, for which large area graphene/h-BN film were reliably obtained. We also varied the CH_4_ (from 0.5 sccm to 10 sccm) and H_2_ (from 5 sccm to 100 sccm) flow rates while keeping the growth time to 20 min, to assess the effects of these manipulations on the growth characteristics. Similar characteristics were found as a function of gas flow rates (Figures S1 and S2 in the supporting information). It should be noted that we also investigated the influence of different growth times on both the Raman spectra characteristics and the FWHM values. For instance, Fig. 5(g),(h) show that the I_2D_/I_G_ intensity ratio is nearly the same (*i.e*., I_2D_/I_G_ > 2) and the FWHM values are quite similar (36–39 cm^−1^). These results also confirm that the grown graphene is a single-layer^22^. Furthermore, the Raman peak characteristics (including the FWHM values) were quite similar for different CH_4_ and H_2_ flow rates (Figures S3 and S4 in the supporting information). These results clearly indicate that the graphene/h-BN heterostructures can be reliably obtained using the LPCVD.

### Scanning electron microscopy (SEM) and high-resolution transmission electron microscopy (HR-TEM) characterizations

**Figure 6:**
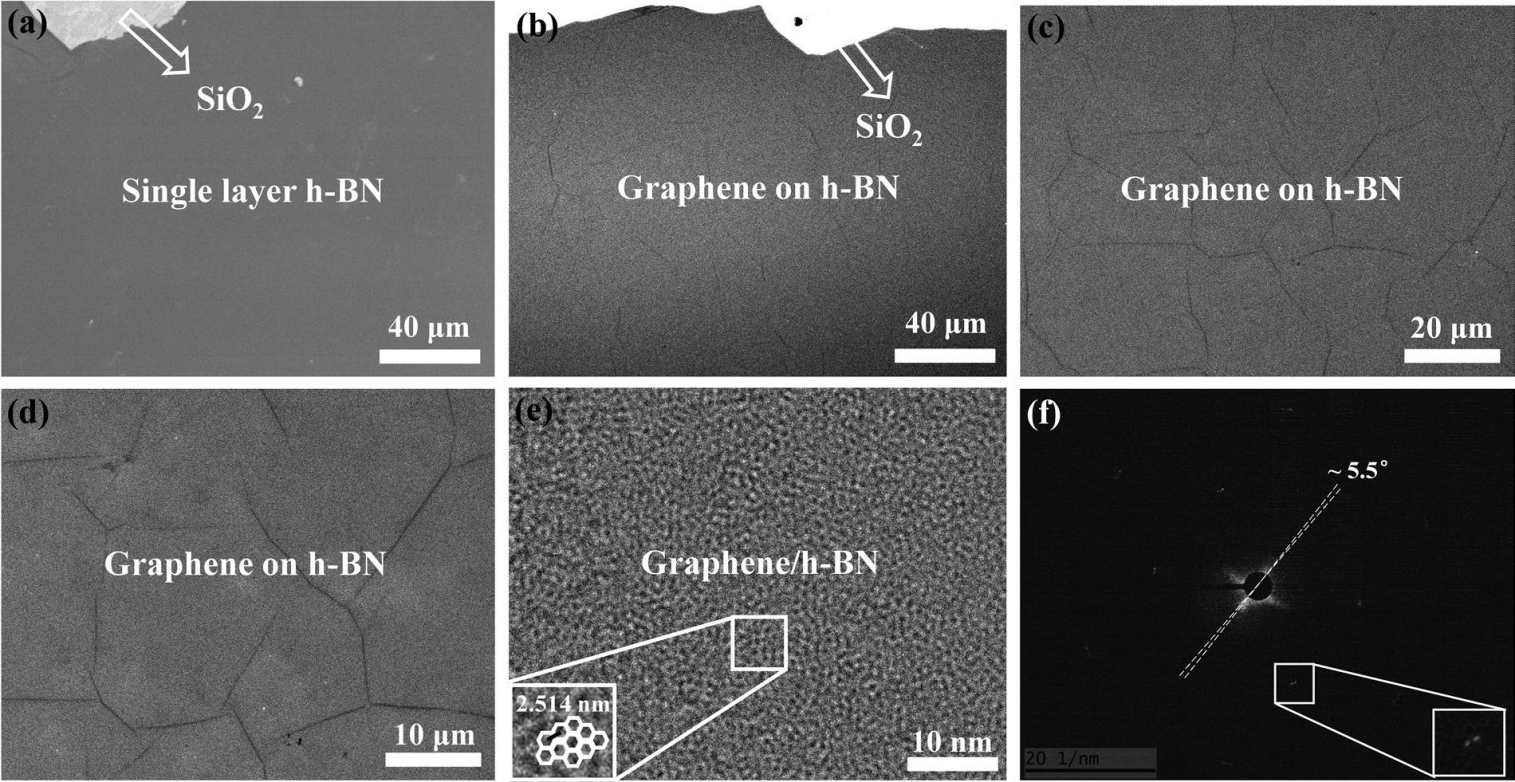
SEM and HR-TEM images of graphene on h-BN. (**a**) Single-layer h-BN. (**b**), (**c**), and (**d**), single-layer graphene on the h-BN film transferred onto a 300-nm-thick SiO_2_/Si substrate. (**e**) HR-TEM image of graphene/h-BN heterostructure. (**f**) The SAED image of graphene/h-BN heterostructure.

Figure 6(a) shows a representative SEM micrograph of a single-layer h-BN transferred onto a 300-nm-thick SiO_2_/Si substrate. The h-BN surface is continuous, which is consistent with the corresponding optical images. The SEM images of the graphene/h-BN film are shown in Fig. 6(b)–(d). From Fig. 6(b), it can be seen that the continuous graphene film is grown on the h-BN film. In particular, as clearly shown in Fig. 6(c),(d), direct growth of graphene on the h-BN film is successfully realized, which not only allows obtaining continuous graphene film, but also forms graphene/h-BN heterostructure for viable device applications. Figure 6(e) shows the HR-TEM image of graphene/h-BN vertical heterostructure. The selective area electron diffraction (SAED) pattern is also shown in Fig. 6(f). As evident from this figure, a clear hexagonal Moiré pattern of graphene/h-BN sample was observed with the rotation angle of about 5.5° and the Moiré pattern period of about 2.514 nm, suggesting that graphene and h-BN are not precisely aligned. However, the SAED patterns revealing symmetric diffraction spots, Raman spectra and electrical transport data, indicate that high quality graphene/h-BN vertical hetero-structure with a rotation angle of 5.5° between graphene and h-BN.

### Atomic Force Microscopy (AFM) characterization

**Figure 7:**
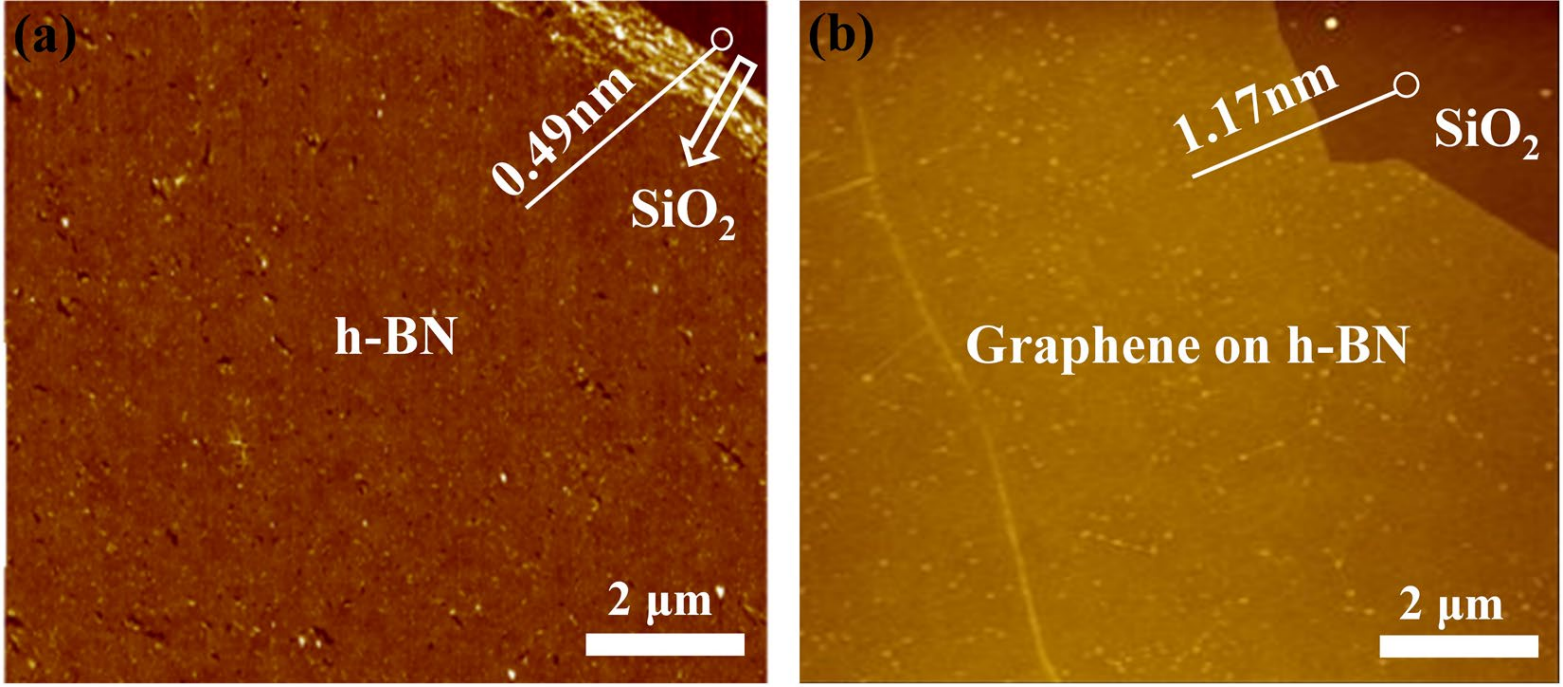
AFM images of samples. (**a**) Single-layer h-BN, and (**b**) graphene on h-BN transferred onto a 300-nm-thick SiO_2_ substrate.

The atomic structure and thickness of the h-BN and graphene/h-BN films were determined using AFM. Figure 7(a) shows the AFM image of the h-BN film, indicating that the h-BN film thickness is about 0.5 nm, which supports our claim that the obtained h-BN film is single-layer^19,23^. Figure 7(b) shows the AFM image of the graphene/h-BN film transferred onto the 300-nm-thick SiO_2_/Si substrate. Based on the analysis performed in Fig. 7(b), the graphene grown directly on the h-BN film exhibits a smooth surface, and the thickness of the graphene/h-BN film is approximately 1.17 nm, revealing that graphene on h-BN film can easily form graphene/h-BN vertical heterostructures.

The CVD-based synthesis of uniform-thickness single layer h-BN films grown on Pt foils with a borazine source has been reported elsewhere^19,24^. A borazine source was catalytically decomposed on the Pt surface, leading to the self-limiting growth of the single-layer without the associating precipitation, which was very similar to the growth of graphene on Cu. The single layer h-BN film was obtained uniformly over the entire Pt foil, regardless of the Pt lattice orientation^19,24^. Besides, h-BN is an exceptional 2-dimensional dielectric material for graphene field effect transistors (FETs), owing to atomically flat and dangling-bond-free h-BN, significantly minimizing the interfacial charge trapping. Moreover, h-BN has a hexagonal structure that represents a typical sp^2^-hybridized material, quite similar to archetypal 2-dimensional graphene^25,26^ (the lattice constants are similar: 2.456 Å for graphene and 2.504 Å for h-BN). The van der Waals epitaxy growth of graphene on h-BN film has been reported by many researchers^15,17,27^. By taking advantage of the single-layer h-BN films grown on Pt foils, our approach to the epitaxial growth of graphene could be very effective for realizing uniform graphene on h-BN films in a controlled manner. Different from the LPCVD growth of graphene on metal foils such as Cu, Ni and Pt, the surface of a metal catalyst plays a key role in the decomposition of gas molecules and the nucleation of absorbed atoms during the growth process. Hence, with the help of catalytic Pt foil covered by the single-layer h-BN film, the decomposition and nucleation sites were effectively controlled. Hence, the CH_4_ gas must have been decomposed owing to the presence of underlying catalytic Pt foil during the growth process. As the growth time increases, the carbon atoms may suffuse swiftly over h-BN film. Additionally, because the h-BN film has a flat surface, with increasing the growth time, the uniform graphene film would be grown on h-BN film. Therefore, the high-quality graphene film can be grown on h-BN/Pt foil through the systematic optimization of growth conditions (CH_4_ flow rate, H_2_ flow rate and growth time). (See Fig. 3(c) and Fig. 5). It should be noted that the h-BN/Pt foil not only possesses controllable catalytic effects but also promotes the high-quality graphene growth on h-BN/Pt foil. Hence, the graphene film can be uniformly grown on the h-BN layer.

### Electrical transport characterization

**Figure 8:**
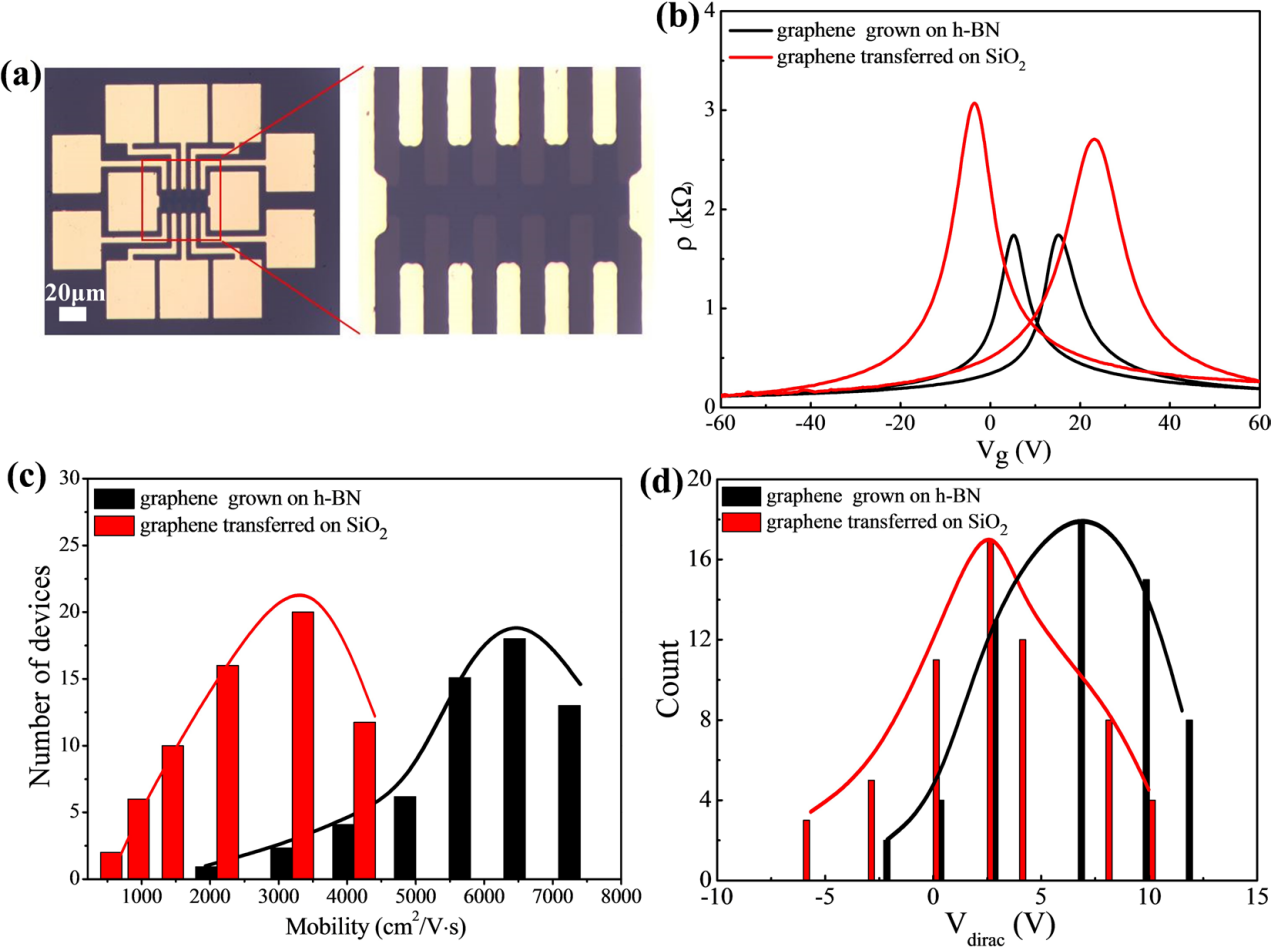
Electrical transport properties of graphene on h-BN. (**a**) Optical microscopic images of the device. (**b**) The longitudinal resistance R *vs*. the applied gate voltage V_gate_, including the graphene grown on h-BN and graphene transferred on SiO_2_. (**c**) Histogram of the carrier mobility distribution of graphene grown on h-BN and graphene transferred on SiO_2_. (**d**) Histogram of the average Dirac point of graphene grown on h-BN and graphene transferred on SiO_2_.

To investigate the electrical transport properties of graphene/h-BN films, we fabricated graphene-based FETs (GFETs) with 40 μm long and 10 μm wide channels by transferring a graphene/h-BN film onto a 100-nm-thick SiO_2_/Si substrate with Cr/Au (20/50 nm) as the source and drain electrodes as shown in Fig. 8(a). The heavily doped p-type Si (~10^20^/cm^3^) substrate was used as a bottom gate that modulates the charge density in the graphene.

Graphene field effect transistors (GFET) by using two different types of graphene grown on h-BN film and transferred on SiO_2_ were prepared to evaluate their electrical characteristics following a standard photolithographic procedure. Figure 8(a),(b) present an optical image of the fabricated GFET device structure and resistivity (ρ) *vs*. gate voltage (*V*_*g*_) curves for both GFET devices, respectively. We obtained the hole mobility of up to 3,900 and 7,200 cm^2^V^−1^s^−1^ and the electron mobility of up to 3,600 and 7,000 cm^2^V^−1^s^−1^, respectively using the simple Drude model^28–30^. Note that the charge carrier mobility values of the graphene grown on h-BN is higher than those of the graphene transferred on SiO_2_. We believe that these observations are attributed to the fact that the intrinsically atomic-flat h-BN not only provides a low number of dangling bonds between graphene and h-BN, but also minimizes graphene’s structural defect density and the doping level owing to its excellent chemical stability and isostructural nature. Therefore, h-BN film is considered an ideal substrate to synthesize high-quality graphene. It should be noted that the mobility values observed in our devices are 2 to 3 times higher than those reported elsewhere for LPCVD grown graphene on h-BN film^31,32^. Moreover, the histogram of the carrier mobility values and the average V_dirac_ for up to 60 devices, shown in Fig. 8(c and d), suggests that the graphene/h-BN heterostructure exhibits high carrier mobility, in the 2,000–8,000 cm^2^V^−1^s^−1^ range. It is important to note that such a high carrier mobility obtained for our graphene/h-BN heterostructures strongly suggests that a direct CVD-based growth of graphene on h-BN using Pt foils is very promising for constructing high-performance graphene-based FETs and tunneling devices, and is advantageous compared with two-step transferred graphene on h-BN film.

We demonstrated direct growth of graphene/h-BN vertical heterostructures on Pt foils using the CVD method. Moreover, we found that using a single-layer h-BN/Pt foil as a substrate not only presents an ideal platform for obtaining continuous graphene/h-BN vertical heterostructures but also enables to realize desirable electrical characteristics of graphene. Direct growth of graphene on h-BN films retains the pristine properties of graphene by avoiding the interfacial contamination, thus yielding a clean interface between the graphene and the h-BN film. On the other hand, because the lattice structures of graphene and h-BN are very similar, during the growth procedure the carbon atoms will suffuse a large area on the h-BN film by diffusion, yielding large-area graphene/h-BN heterostructures. Our graphene/h-BN heterostructure exhibits high carrier mobility, in the 2,000–8,000 cm^2^V^−1^s^−1^ range. Such a high carrier mobility obtained for our graphene/h-BN heterostructures strongly suggests that such graphene/h-BN heterostructures on recyclable Pt foils grown by LPCVD can be a promising approach for high-performance graphene-based electronics.

## Methods

### LPCVD growth of single-layer graphene on h-BN/Pt foil

The growth process was implemented by employing a Pt foil and CH_4_ as the substrate and the carbon precursor carrier, respectively. A single-layer h-BN on the Pt foil was grown using the LPCVD method following our previously developed synthetic method that was reported elsewhere^19^. In addition, to avoid detrimental etching of h-BN films by H-containing gaseous species during the growth of graphene, a 1.5 m long quartz tube furnace with moving rails, allowing easy positioning of the furnace (*i.e*. heating zone) over some length of the quartz tube. Figure 1 shows a schematic of the LPCVD system for growing high-quality large-area graphene on h-BN/Pt foils. A subsequent growth process for obtaining high-quality single-layer graphene on a single-layer h-BN on a Pt foil was as follows: The h-BN/Pt foil was first placed in the furnace region labelled “B” which was kept at room temperature; the furnace region labelled “A” was then heated up using Ar and H_2_ gas flows from room temperature to 1000 °C for 25 min; when the temperature reached 1000 °C, the furnace was then repositioned from “A” to “B” to avoid H_2_ etching during the growth of graphene over the h-BN film. It should be noted that h-BN/Pt foil was kept at room temperature during the process of the furnace heating and was only exposed right after the temperature reaches 1000 °C. This is a key step for avoiding H_2_ etching as we can minimize the reaction time to etch away h-BN during the graphene growth. The Ar gas flow was then turned off and gaseous CH_4_ was introduced; the growth of graphene continued for 20 min; after the growth, H_2_ was turned off and the furnace was moved from “B” to “A” to allow a natural cool down to room temperature. The optimization of growth parameters for obtaining high-quality large-area single-layer graphene on the h-BN/Pt foil was systematically performed by varying the H_2_ and CH_4_ flow rate as well as the growth time.

### Transfer of graphene/h-BN film using the electrochemical bubbling method

Figure S1 schematically shows the electrochemical bubbling method-based transfer of a graphene/h-BN film onto a 300-nm-thick SiO_2_/Si substrate. First, the graphene/h-BN/Pt foil was spin-coated with polyvinyl alcohol (PVA) and poly methyl methacrylate (PMMA) layers. The sample was then kept in vacuum for 24 hr. After establishing the vacuum, the electrochemical bubbling method was used for transferring PMMA/PVA/graphene/h-BN onto a 300-nm-thick SiO_2_/Si substrate. The PMMA/PVA/graphene/h-BN/Pt foil and a pure Pt foil were used as the cathode and the anode, respectively; a NaOH aqueous solution (1.0 M) was used as the electrolyte at room temperature. The bubbling transfer method was used with a stable current of 1 A, and the corresponding electrolytic voltage was run between 5–10 V and was applied for 2–5 min. After the PMMA/PVA/graphene/h-BN film was peeled off from the Pt foil, the sample was cleaned 3 times using deionized water to remove the residual NaOH solution, after which the sample was transferred onto a 300-nm-thick SiO_2_/Si substrate. Finally, the sample was dipped in acetone for 10 min to remove PMMA and then kept in hot water (100 °C) for 15 min to remove PVA.

### Characterizations

Optical micrographs were acquired using optical microscope (Olympus, Olympus DX51). The surface morphology was measured using field-emission scanning electron microscope (JEOL JSM7401F) and transmission electron microscopy (JEOL 2100 F, 200 kV). The film thickness and the surface topography were measured using an atomic force microscope (Veeco, Dimension 3100). Raman spectra were measured using a micro Raman microscope (Renishaw, InVia Basic) with a 532-nm-wavelength laser.

## Electronic supplementary material

Supporting Information

## References

[1] Ci L (2010). Atomic layers of hybridized boron nitride and graphene domains. Nature materials.

[2] Tang S (2015). Silane-catalysed fast growth of large single-crystalline graphene on hexagonal boron nitride. Nature communications.

[3] Han G (2013). Continuous growth of hexagonal graphene and boron nitride in-plane heterostructures by atmospheric pressure chemical vapor deposition. ACS Nano.

[4] Roth S, Matsui F, Greber T, Osterwalder J (2013). Chemical vapor deposition and characterization of aligned and incommensurate graphene/hexagonal boron nitride heterostack on Cu(111). Nano letters.

[5] Xue J (2011). Scanning tunnelling microscopy and spectroscopy of ultra-flat graphene on hexagonal boron nitride. Nature materials.

[6] Kretinin AV (2014). Electronic properties of graphene encapsulated with different two-dimensional atomic crystals. Nano letters.

[7] Woods CR (2014). Commensurate–incommensurate transition in graphene on hexagonal boron nitride. Nature Physics.

[8] Zeng Q (2015). Band engineering for novel two-dimensional atomic layers. Small.

[9] Cobaleda C, Pezzini S, Diez E, Bellani V (2014). Temperature-and density-dependent transport regimes in ah-BN/bilayer graphene/h-BN heterostructure. Phys. Rev. B.

[10] Kim KK (2012). Synthesis and characterization of hexagonal boron nitride film as a dielectric layer for graphene devices. ACS Nano.

[11] Yankowitz M (2012). Emergence of superlattice Dirac points in graphene on hexagonal boron nitride. Nature Physics.

[12] Mayorov AS (2011). Micrometer-scale ballistic transport in encapsulated graphene at room temperature. Nano letters.

[13] Dean CR (2010). Boron nitride substrates for high-quality graphene electronics. Nature nanotechnology.

[14] Haigh SJ (2012). Cross-sectional imaging of individual layers and buried interfaces of graphene-based heterostructures and superlattices. Nature materials.

[15] Yang W (2013). Epitaxial growth of single-domain graphene on hexagonal boron nitride. Nature materials.

[16] Liu Z (2011). Direct growth of graphene/hexagonal boron nitride stacked layers. Nano letters.

[17] Zhang C (2015). Direct growth of large-area graphene and boron nitride heterostructures by a co-segregation method. Nature communications.

[18] Gao L (2012). Repeated growth and bubbling transfer of graphene with millimetre-size single-crystal grains using platinum. Nature communications.

[19] Kim G (2013). Growth of high-crystalline, single-layer hexagonal boron nitride on recyclable platinum foil. Nano letters.

[20] Kim KK (2012). Synthesis of monolayer hexagonal boron nitride on Cu foil using chemical vapor deposition. Nano letters.

[21] Ferrari AC (2006). Raman spectrum of graphene and graphene layers. Physical review letters.

[22] Malard LM, Pimenta MA, Dresselhaus G, Dresselhaus MS (2009). Raman spectroscopy in graphene. Physics Reports.

[23] Kim SM (2015). Synthesis of large-area multilayer hexagonal boron nitride for high material performance. Nature communications.

[24] Park JH (2014). Large-area monolayer hexagonal boron nitride on Pt foil. ACS Nano.

[25] Lee KH (2012). Large-scale synthesis of high-quality hexagonal boron nitride nanosheets for large-area graphene electronics. Nano letters.

[26] Ahn G (2013). Optical probing of the electronic interaction between graphene and hexagonal boron nitride. ACS Nano.

[27] Mishra N (2016). Rapid and catalyst-free van der Waals epitaxy of graphene on hexagonal boron nitride. Carbon.

[28] Van NH, Qian Y, Han SK, Kang DJ (2016). PMMA-etching-free transfer of wafer-scale chemical vapor deposition two-dimensional atomic crystal by a water soluble polyvinyl alcohol polymer method. Scientific reports.

[29] Lee JH (2014). Wafer-scale growth of single-crystal monolayer graphene on reusable hydrogen-terminated germanium. Science.

[30] Das A (2008). Monitoring dopants by Raman scattering in an electrochemically top-gated graphene transistor. Nature nanotechnology.

[31] Li X (2015). Noise in graphene superlattices grown on hexagonal boron nitride. ACS Nano.

[32] Gong Y (2014). Direct chemical conversion of graphene to boron- and nitrogen-and carbon-containing atomic layers. Nature communications.

