# Supplementary material for: Growth of Graphene/h-BN Heterostructures on Recyclable Pt Foils by One-Batch Chemical Vapor Deposition
Source: Sci Rep. 2017 Dec 6;7:17083. doi: 10.1038/s41598-017-17432-9 (PMC5719054; doi:10.1038/s41598-017-17432-9)
Supplement: Supplementary file 1 — Supporting Information [file 41598_2017_17432_MOESM1_ESM.doc]

**Supporting Information**

Growth of Graphene/h-BN Heterostructures on Recylable Pt Foils by One-Batch Chemical Vapor Deposition

Yongteng Qian, Huynh Van Ngoc, Dae Joon Kang*

Department of Physics and Interdisciplinary Course of Physics and Chemistry, Sungkyunkwan University, 2066 Seobu-ro, Jangan-gu, Suwon 16419, Gyeonggi-do, Republic of Korea.

^＊^Author to whom the correspondence should be made : djkang@skku.edu (+82-31-290-5906)

**Optical micrographs of graphene/h-BN at different CH_4_ flow rates**


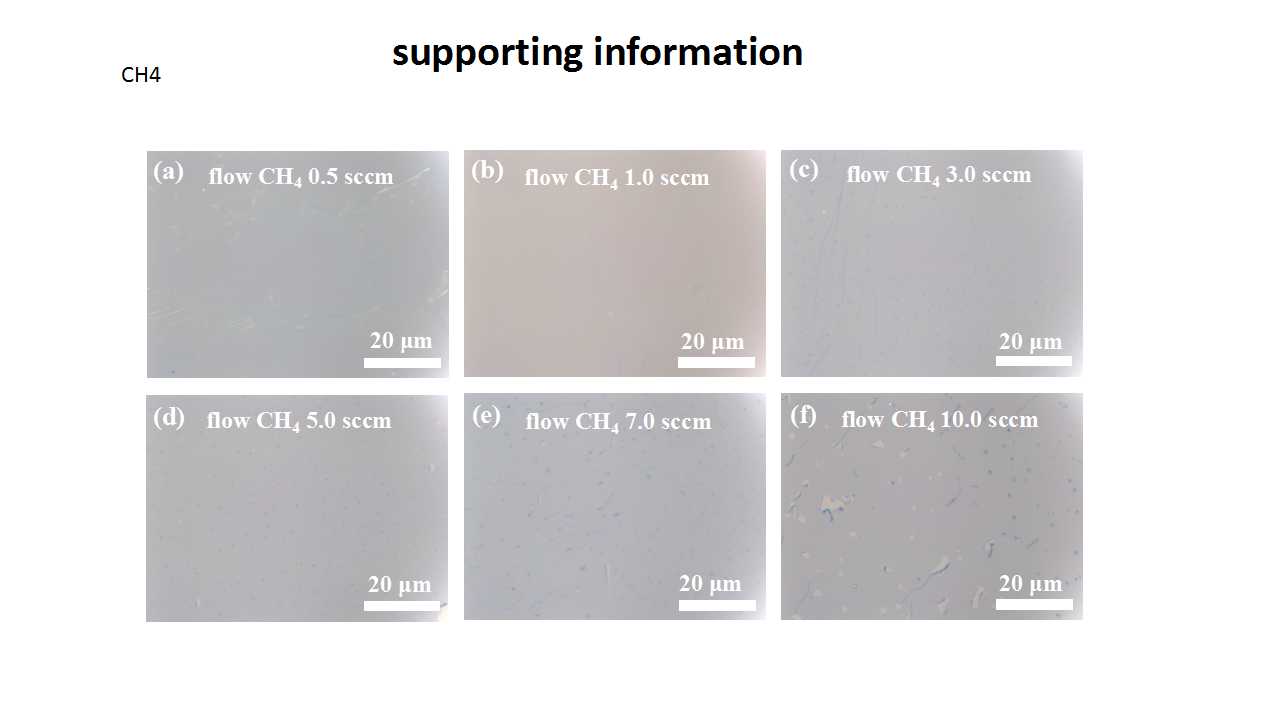


**Figure S1.** Optical micrographs of graphene/h-BN film transferred onto a 300-nm-thick SiO_2_/Si substrate. (a-f) Optical micrographs of graphene/h-BN film grown at different CH_4_ flow rates: (a) 0.5 sccm, (b) 1.0 sccm, (c) 3.0 sccm, (d) 5.0 sccm, (e) 7.0 sccm, and (f) 10.0 sccm, respectively. These optical micrographs reveal that the surface of graphene/h-BN film is found to be discontinuous for the CH_4_ flow rates of (a) 0.5 sccm and (f) 10.0 sccm. For the CH_4_ flow rates of (b) 1.0 sccm and (c) 3.0 sccm, the surface of graphene/h-BN film were uniform and continuous. On the other hand, for the CH_4_ flow rates of (d) 5.0 sccm and (e) 7.0 sccm, numerous particulate defects were observed on the surface. Based on these observations, we concluded that the optimal CH_4_ flow rate is 1.0 sccm.

**Optical micrographs of graphene/h-BN at different H_2_ flow rates**

**
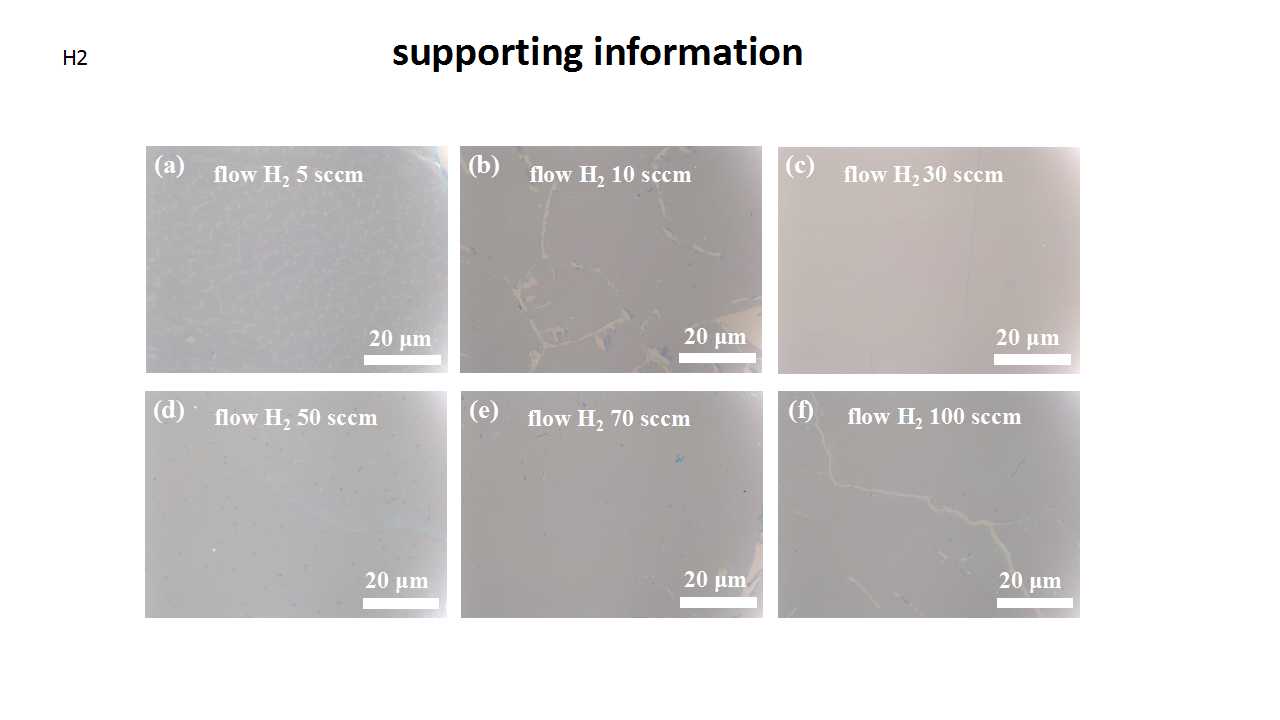
**

**Figure S2.** Optical micrographs of graphene/h-BN film transferred onto a 300-nm-thick SiO_2_/Si substrate. (a-f) Optical micrographs of graphene/h-BN film grown at different H_2_ flow rates: (a) 5 sccm, (b) 10 sccm, (c) 30 sccm, (d) 50 sccm, (e) 70 sccm, and (f) 100 sccm, respectively. These optical micrographs reveal that graphene/h-BN film is discontinuous for the H_2_ flow rates of (a) 5 sccm, (b) 10 sccm, (e) 70 sccm and (f) 100 sccm; For the H_2_ flow rate of 30 sccm, the surface of graphene/h-BN film was found to be continuous over a large area. On the other hand, numerous particulate defects are observed for the H_2_ flow rate of (d) 50 sccm. We therefore concluded that the optimum H_2_ flow rate is 30 sccm.

**Raman spectra and FWHM of graphene/h-BN at different CH_4_ flow rates**

**
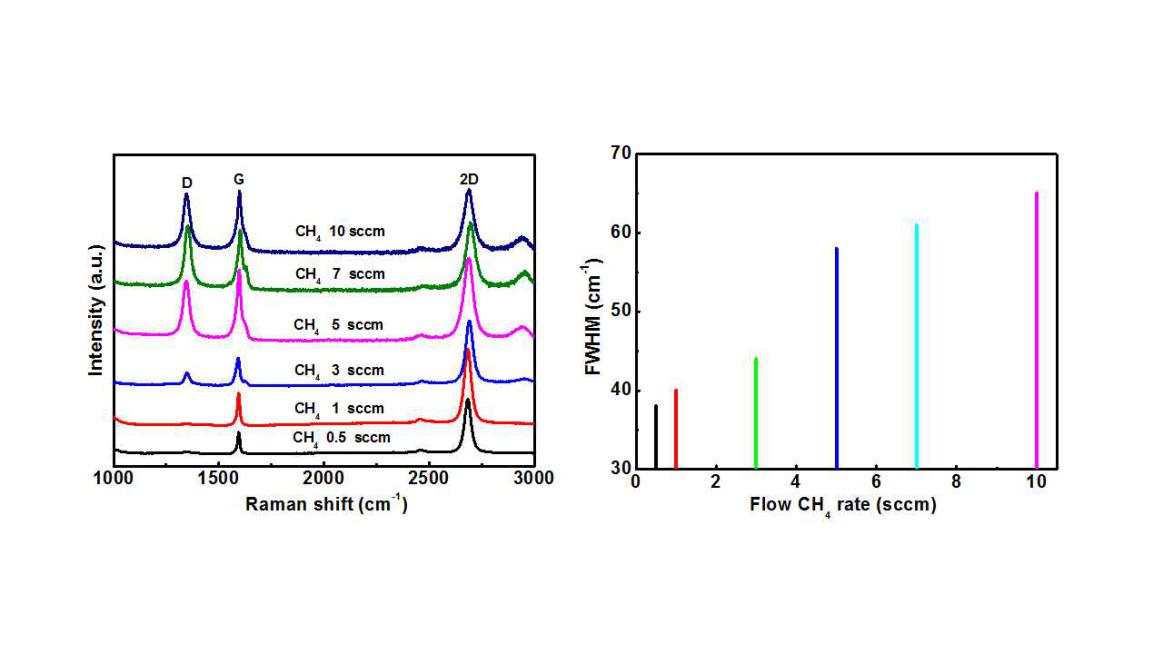
**

**(a)**

**(b)**

**Figure S3.** Raman spectra of graphene/h-BN film transferred onto a 300-nm-thick SiO_2_/Si substrate. (a) A series of Raman spectra of graphene/h-BN film at different CH_4_ flow rates varying from 0.5 to 10 sccm. (b) FWHM of the 2D band of graphene/h-BN film at different CH_4_ flow rates varying from 0.5 to 10 sccm. As shown in Figure S3 (a), when CH_4_ flow rates are 0.5, 1 and 3 sccm, the intensity ratio I_2D_/I_G_ > 2, which indicates a single layer graphene/h-BN film. On the other hand, with increasing the CH_4_ gas flow rates (from 5 to 10 sccm), the intensity ratio I_2D_/I_G_ became around 1, suggesting that the graphene/h-BN film was composed of multiple layers [1]. Based on the analysis of Figure S3 (a), we concluded that the optimum CH_4_ flow rate is 1 sccm, which is also consistent with our optical micrographs as shown in Figure S1.

**Raman spectra and FWHM of graphene/h-BN at different H_2_ flow rates**

**(b)**

**(a)**


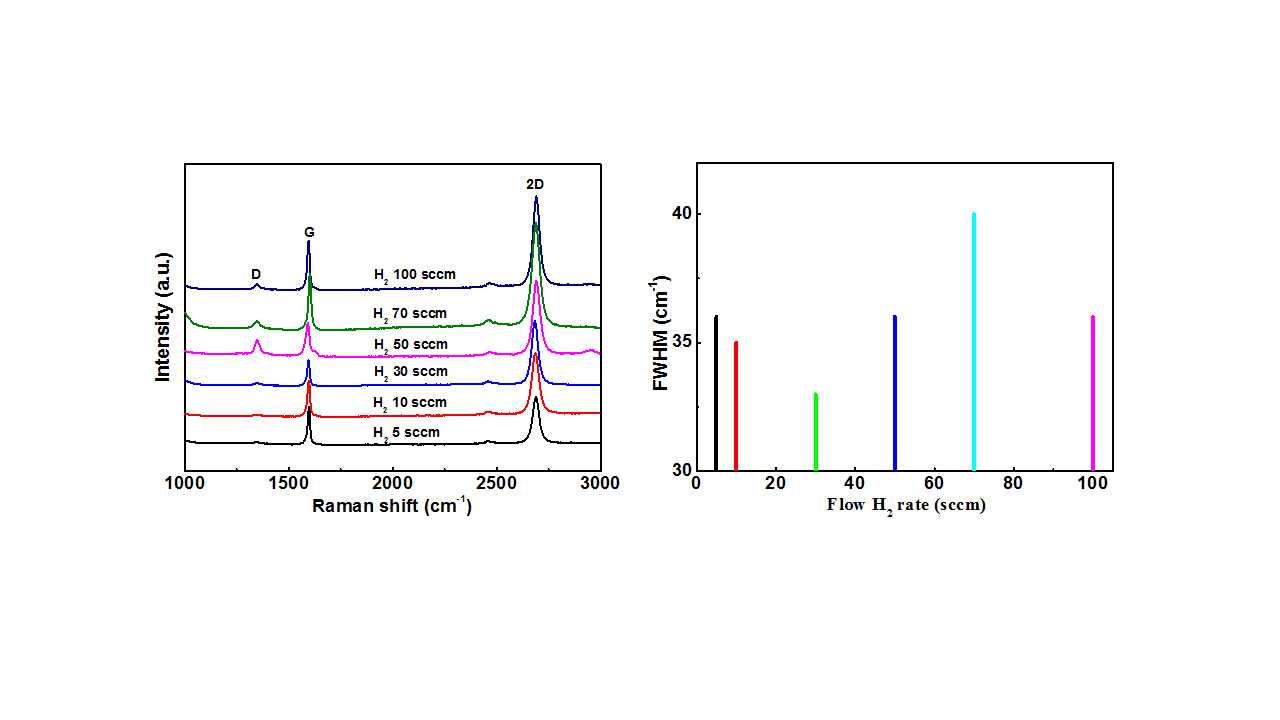


**Figure S4.** Raman spectra of graphene/h-BN film transferred onto a 300-nm-thick SiO_2_/Si substrate. (a) A series of spectrum of graphene/h-BN film at different H_2_ flow rates varying from 5 to 100 sccm. (b) FWHM of the 2D band of graphene/h-BN film at different H_2_ flow rates varying from 5 to 100 sccm. Figure S4 (a) indicates that with increasing H_2_ flow rate (from 5 to 100 sccm), the intensity ratio I_2D_/I_G_ value appears as a kind of parabolic shape (1.5－3－1.5). More importantly, when H_2_ gas flow rate is 30 sccm, the I_2D_/I_G_ has an optimum value (I_2D_/I_G_ >2). Based on the analysis of Figure S4 (a), we concluded that the optimum H_2_ gas flow rate is 30 sccm, which is also consistent with the optical micrographs shown in Figure S2.

References

1. Zhao, P. et al. Equilibrium chemical vapor deposition growth of bernal-stacked bilayer graphene. ACS Nano **11**, 11631-11638 (2014).
